# Intramural duodenal hematoma following therapeutic endoscopy: a case report of a rare adverse event

**DOI:** 10.1016/j.igie.2025.10.007

**Published:** 2025-10-20

**Authors:** Bryan W. Ferrigno, Katharine A. Germansky

**Affiliations:** 1 Department of Gastroenterology, Beth Israel Deaconess Medical Center, Boston, Massachusetts, USA; 2 Harvard Medical School, Boston, Massachusetts, USA

## Abstract

An intramural duodenal hematoma (IDH) is an uncommon clinical entity that can be an adverse event of therapeutic esophagogastroduodenoscopy (EGD). Herein, we present a case of a 60-year-old man who presented to the hospital with vomiting, diarrhea, and overt gastrointestinal bleeding with melena and small-volume hematemesis. EGD was pursued and revealed a bleeding duodenal ulcer that was therapeutically intervened upon to achieve hemostasis. After endoscopy, his hospital course was complicated by an intramural duodenal hematoma, which presented with abdominal pain and worsening anemia without recurrent overt gastrointestinal bleeding. After consultation with interventional radiology and surgery, he ultimately improved with supportive care and was discharged home without further endoscopic or procedural intervention. An IDH is an uncommon gastrointestinal pathology that should be considered after endoscopy in the setting of abdominal pain and anemia without overt bleeding. Conservative management often leads to successful outcomes, but multidisciplinary care should be considered in the setting of further adverse events.

## Introduction

An intramural duodenal hematoma (IDH) is an uncommon gastrointestinal pathology, classically associated with abdominal trauma, thought to be due to the duodenum's relatively fixed retroperitoneal position. Nontraumatic causes such as coagulopathies, pancreatic disorders, malignancies, and procedural adverse events—including endoscopy—remain rare but are becoming more widely recognized.^1^, ^2^, ^3^ We present the case of an IDH as a rare adverse event of therapeutic esophagogastroduodenoscopy (EGD).

## Case description

Our case involves a 60-year-old man with a medical history of pulmonary hypertension, congestive heart failure, alcohol use disorder, and iron deficiency anemia who was admitted to the hospital with several gastrointestinal symptoms. He initially presented to the emergency department with 1 day of nausea, vomiting, and diarrhea. He also reported a single episode of blood-streaked emesis as well as streaks of bright red blood in the stool. He was not on antiplatelet or anticoagulant medications. He did not have any pertinent family history. Pertinent social history included a history of alcohol use disorder, averaging 20 to 25 drinks per week. Physical examination results were unremarkable except for mild left lower quadrant tenderness.

The patient was found to have a lactic acidosis with a lactate level of 6.4 mmol/L (normal range, 0.5-2.0 mmol/L) when he first arrived, as well as anemia with a hemoglobin level of 5.6 g/dL, decreased from a prior baseline of 12.0 g/dL 3 months prior (normal range, 14-17 g/dL). The international normalized ratio was normal at 1.1 (normal range, 0.8-1.2). He had a history of chronic thrombocytopenia with a platelet count of 60,000/μL (normal range, 150K-400K/μL), thought to be due to alcohol use disorder. He was found to have an acute kidney injury with a creatinine level of 4.9 mg/dL, increased from a baseline of 1.0 mg/dL 3 months prior (normal range, 0.6-1.30 mg/dL), and a blood urea nitrogen (BUN) level of 148 mg/dL (normal range, 6-20 mg/dL). He was hypotensive despite fluid resuscitation and transfusion of 2 units of packed red blood cells and was admitted to the intensive care unit for further supportive care including vasopressor initiation. He did not have any overt gastrointestinal bleeding until hospital day 2, when he developed melena.

**Figure 1:**
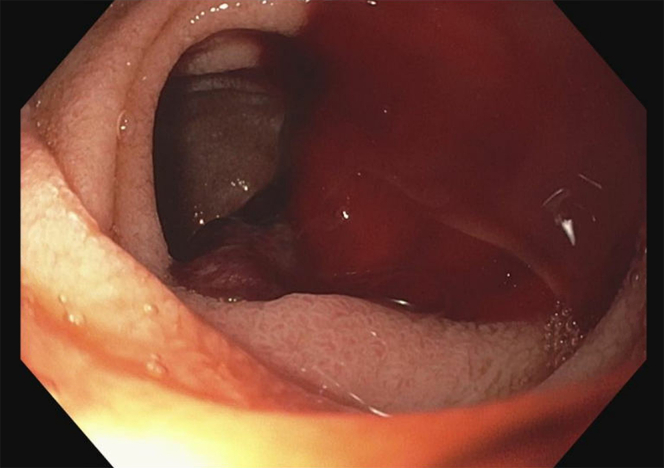
Esophagogastroduodenoscopy with blood and a blood clot in the second part of the duodenum.

**Figure 2:**
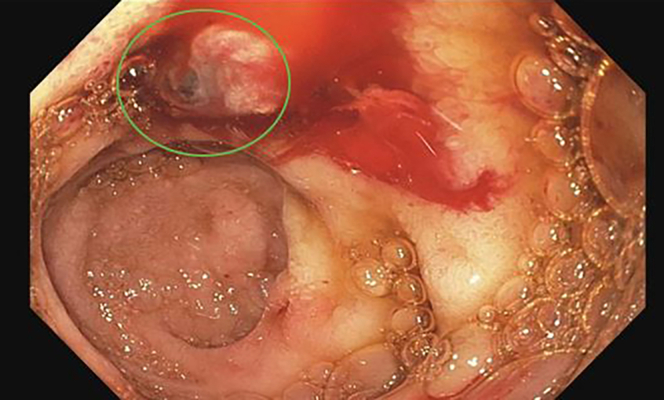
Duodenal ulcer and visible vessel (*green circle*) in the second part of the duodenum.

**Figure 3:**
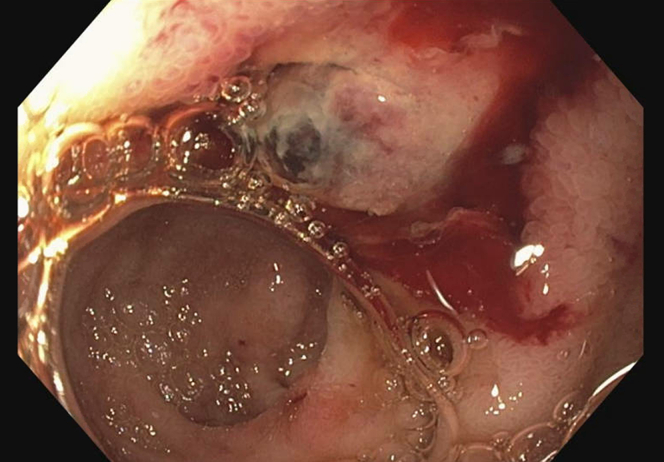
Duodenal ulcer after epinephrine injection.

**Figure 4:**
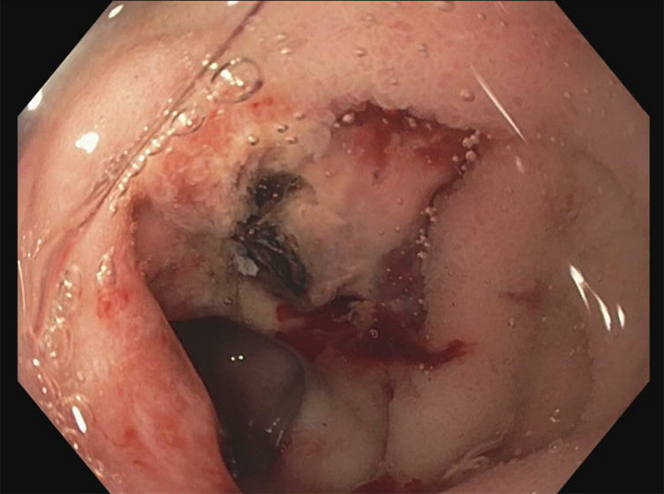
Duodenal ulcer after epinephrine injection and bipolar circumactive probe (Boston Scientific Marlborough, Mass, USA) electrocautery.

He underwent EGD (Olympus GIF-HQ190, Center Valley, Pa, USA) on hospital day 2, which revealed an actively bleeding 1-cm ulcer in the second part of the duodenum (Forrest class Ib) with an overlying clot and a visible vessel (Figs. 1 and 2). Endoscopic clip placement was attempted (Resolution 360 ULTRA Clip; Boston Scientific, Marlborough, Mass, USA); the clip dislodged the clot but ultimately fell off of the mucosa. Epinephrine 0.1 mg/mL was subsequently injected into the submucosa, followed by bipolar circumactive probe (Boston Scientific) electrocautery with hemostasis achieved (Figs. 3 and 4). His hemoglobin level before the procedure was 7.5 g/dL (Table 1).

**Table 1:** Trend of the patient's hemoglobin and clinical events across hospitalization

Hospital day	Hemoglobin (g/dL)	Clinical event
Preadmission 3 months prior	12	Asymptomatic
Day 0	5.6	Blood-streaked emesis, blood-streaked stool; transfused 2U pRBCs
Day 1	8.0	In the ICU, requiring vasopressors; no overt bleeding
Day 2	7.5	Developed melena, underwent EGD
Day 3	6.6 in the morning; 8.3 in the evening after transfusion	Transfused 1U pRBCs, developed abdominal pain
Day 4	6.6	CTA AP performed, diagnosing an IDH; transfused 1U pRBCs
Day 5	7.9	NPO
Day 6	8.7	Diet advanced to clear liquids
Day 7	8.9	Diet advanced to regular
Day 8	9.1	Discharged home

*CTA AP*, Computed tomography angiography of the abdomen and pelvis; *EGD*, esophagogastroduodenoscopy; *ICU*, intensive care unit; *IDH*, intramural duodenal hematoma; *NPO*, nil per os; *pRBCs*, packed red blood cells; *U*, units.

On hospital day 3, his hemoglobin level dropped from 7.5 g/dL to 6.6 g/dL, and he was transfused with an appropriate response in hemoglobin (Table 1). He had no recurrent overt bleeding; bowel movements were brown. On the evening of hospital day 3, he reported abdominal pain.

**Figure 5:**
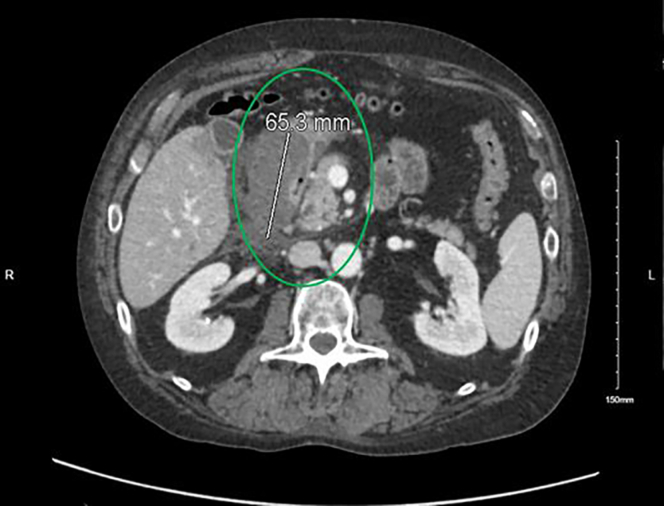
Computed tomography angiography axial view of the intramural duodenal hematoma *(green circle)*.

**Figure 6:**
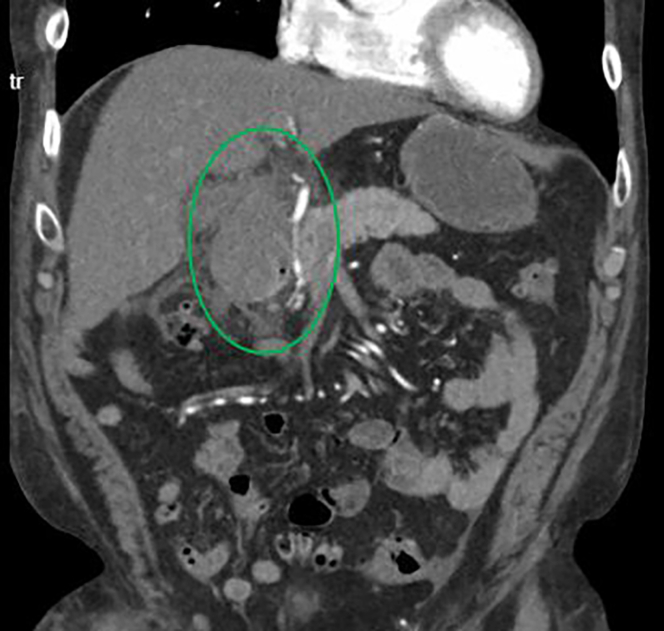
Computed tomography angiography coronal view of the intramural duodenal hematoma *(green circle)*.

On hospital day 4, his hemoglobin level again dropped from 8.3 to 6.6 g/dL, and he received an additional blood transfusion (Table 1). His abdominal pain worsened. Repeat endoscopic evaluation was considered, but given severe abdominal pain and lack of recurrent overt bleeding, computed tomography with angiography of the abdomen and pelvis (CTA AP) was pursued. CTA AP revealed evidence of an intramural hematoma in the descending duodenum without active extravasation, measuring 6.3 × 5.7 × 4.4 cm (Figs. 5 and 6). CTA AP additionally noted a right vastus intermedius intramuscular hematoma measuring 9.3 × 6.9 cm, without active extravasation.

General surgery and interventional radiology services were consulted for the duodenal hematoma. Ultimately, conservative management was pursued with nil per os (NPO) for 24 hours, followed by slow dietary advancement, supportive intravenous fluids, and continued antisecretory therapy with proton pump inhibitors. The patient was eager to leave the hospital, and he was ultimately discharged on hospital day 8. His BUN and creatinine were within normal limits at hospital discharge. He was subsequently lost to follow-up, and thus we were unable to solicit his perspective on the care he received.

## Discussion

An IDH is an uncommon gastrointestinal pathology, classically associated with abdominal trauma, thought to be due to the duodenum's relatively fixed retroperitoneal position. In this case, the development of an IDH was suspected to be an adverse event after therapeutic EGD. An IDH is an uncommon adverse event of therapeutic EGD and has more commonly been reported in patients with coagulopathies or on antiplatelet or anticoagulation medications—this is consistent with our case presentation given underlying thrombocytopenia.^1^ Our patient also notably had an intramuscular thigh hematoma, further suggestive of sequelae of thrombocytopenia with possible underlying platelet dysfunction.

Endoscopic procedures, particularly those involving manipulation of the duodenal wall such as biopsy, cautery, or injection of submucosal agents, may cause injury to the rich submucosal vascular plexus within the duodenal wall. Furthermore, because the second and third parts of the duodenum are relatively fixed, it may be more prone to shearing injury and trauma from the endoscope.^4^ Our patient, who was undergoing treatment for a duodenal ulcer with high-risk stigmata, underwent multiple interventions during EGD including endoscopic clip placement, epinephrine injection, and BICAP electrocautery. Each of these interventions, although essential to achieve hemostasis, poses a risk of localized trauma to the duodenal wall, which can lead to the development of the hematoma.

In the setting of an acute IDH, the patient's clinical presentation can be variable but often includes symptoms of abdominal pain, nausea, vomiting, and anemia without overt gastrointestinal bleeding. Small-bowel and biliary obstruction due to the mass effect of the hematoma are possible, but rare.^5^^,^^6^

The diagnostic approach to an IDH typically involves imaging, with contrast-enhanced computed tomography or computed tomography with angiography being the preferred modalities; ultrasound can also be considered. Early recognition is critical, because prompt diagnosis can prevent unnecessary interventions and guide appropriate management.

Management of IDHs depends on the clinical severity but is typically conservative. Most patients respond well to bowel rest, supportive fluids, and correction of any underlying coagulopathies. In this case, after consultation with surgical and interventional radiology teams, conservative treatment with NPO status, gradual dietary advancement, and proton pump inhibitor therapy was implemented. The patient showed clinical improvement and was discharged after an uneventful recovery. Surgery or interventional radiology procedures are reserved for cases of ongoing bleeding, obstruction, perforation, or other adverse events. Endoscopic management with drainage of the hematoma using lumen-apposing stents has also been reported.^7^ A limitation of our case report is that our patient was lost to follow-up; therefore, we have no long-term information on how he did with the hematoma.

This case highlights the importance of considering IDHs in patients presenting with abdominal pain and anemia after therapeutic endoscopy, particularly in the absence of overt gastrointestinal bleeding. It also underscores the utility of CTA in the evaluation of postendoscopic adverse events. Although rare, IDHs should be part of the differential diagnosis in similar clinical settings, because timely and appropriate management is critical. Conservative management with bowel rest, gradual dietary advancement, and supportive care often leads to successful outcomes. Awareness of this uncommon adverse event is key for any endoscopist who performs therapeutic or diagnostic interventions during EGD.

## Patient Consent

The patient in this article has given written informed consent to publication of their case details.

## Disclosure

The following author disclosed financial relationships: K. A. Germansky: Grant funding from Exact Sciences. The other author disclosed no financial relationships.
